# Drivers of hospitalization cost after craniotomy for tumor resection: creation and validation of a predictive model

**DOI:** 10.1186/s12913-015-0742-2

**Published:** 2015-03-04

**Authors:** Symeon Missios, Kimon Bekelis

**Affiliations:** Department of Neurosurgery, Cleveland Clinic, Cleveland, OH USA; Section of Neurosurgery, Dartmouth-Hitchcock Medical Center, Lebanon, NH USA

**Keywords:** Brain tumor, Craniotomy for tumor resection, Drivers of cost, Cost prediction, NIS

## Abstract

**Background:**

The economic sustainability of all areas of medicine is under scrutiny. Limited data exist on the drivers of cost after a craniotomy for tumor resection (CTR). The objective of the present study was to develop and validate a predictive model of hospitalization cost after CTR.

**Methods:**

We performed a retrospective study involving CTR patients who were registered in the Nationwide Inpatient Sample (NIS) database from 2005–2010. This cohort underwent 1:1 randomization to create derivation and validation subsamples. Regression techniques were used for the creation of a parsimonious predictive model.

**Results:**

Of the 36,433 patients undergoing CTR, 14638 (40.2%) underwent craniotomies for primary malignant, 9574 (26.3%) for metastatic, and 11414 (31.3%) for benign tumors. The median hospitalization cost was $24,504 (Interquartile Range (IQR), $4,265-$44,743). Common drivers of cost identified in the multivariate analyses included: length of stay, number of procedures, hospital size and region, and patient income. The models were validated in independent cohorts and demonstrated final R^2^ very similar to the initial models. The predicted and observed values in the validation cohort demonstrated good correlation.

**Conclusions:**

This national study identified significant drivers of hospitalization cost after CTR. The presented model can be utilized as an adjunct in the cost containment debate and the creation of data-driven policies.

**Electronic supplementary material:**

The online version of this article (doi:10.1186/s12913-015-0742-2) contains supplementary material, which is available to authorized users.

## Background

The recent seismic changes in US healthcare are driven by the push for economic sustainability of the system [1,2]. Several value-based initiatives aim to minimize cost in areas of increased spending and promote rationalization of resource allocation [1]. Neurosurgical procedures are associated with significant risks and high hospitalization costs. Craniotomy for tumor resection (CTR) is one of the most common such procedures, and will be part of the cost containment debate. The estimation of the hospitalization cost for each individual CTR patient, and the identification of modifiable drivers of cost could allow physicians to understand the economic aspects of CTR, and modify their practice accordingly. Future attempts at cost containment could focus on these factors, rather than follow an arbitrary path.

Several studies have analyzed the cost-effectiveness of different treatment modalities for brain tumors [3-10]. Others have examined the cost or charges of the hospitalization after CTR [11,12]. The latter have limited generalization since they are referring to single institutions or regional experiences, demonstrating significant selection bias. There is a paucity of national data on the hospitalization cost of patients undergoing CTR, the drivers of this cost, and predictive models at the level of the individual patient.

The National Inpatient Sample (NIS) [13] is an all payer, hospital discharge database that represents approximately 20% of all inpatient admissions to nonfederal hospitals in the United States. It allows the unrestricted study of the patient population in question. Using this database, several socioeconomic variables, as well as patient and hospital level factors associated with cost variability after CTR were identified. Based on these data, a predictive model of cost after CTR was developed and validated in an independent cohort.

## Methods

### National Inpatient Sample (NIS) Database

All patients undergoing CTR, who were registered in the National Inpatient Sample (NIS) [13] Database (Healthcare Cost and Utilization Project, Agency for Healthcare Research and Quality, Rockville, MD) between 2005 and 2010, were included in the analysis. The NIS is an all-payer prospective hospital discharge database that represents approximately 20% of all inpatient admissions to nonfederal hospitals in the US. More information about the NIS is available at http://www.ahcpr.gov/data/hcup/nisintro.htm. This database contains de-identified data (consents cannot be obtained), and has been deemed exempt from IRB approval.

### Cohort definition

**Figure 1:**
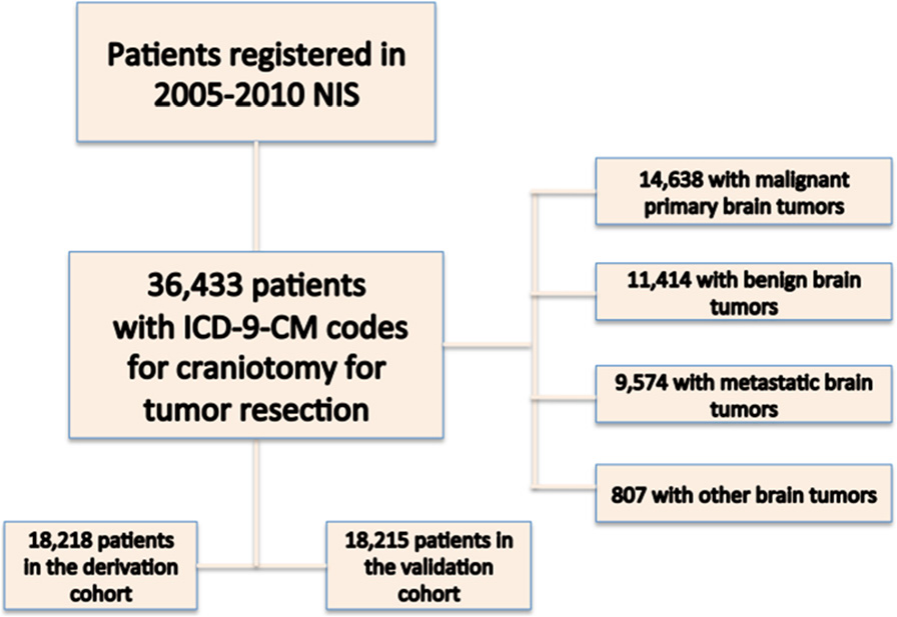
**Cohort selection for the study.**

In order to establish the cohort of patients, we used *International Classification of Disease-9-Current Modification* (ICD-9-CM) codes to identify patients in the registry who underwent craniotomies (ICD-9-CM procedure code 01.51, 01.53, 0.59) for brain tumors (ICD-CM diagnostic code 191.0, 191.1, 191.2, 191.3, 191.4, 191.5, 191.6, 191.7, 191.8, 191.9, 225.0, 225.1, 225.2, 237.5, 237.6, 237.6, 192.0, 192.1, 198.3, 200.5) between 2005 and 2010 (Figure 1). Patients on whom care was withdrawn, or died during the hospitalization were excluded from the cohort.

### Outcome variable

The primary outcome variable was the total hospitalization cost after CTR. Cost data were obtained by conversion of the hospital charges using the group-average cost-to-charge ratio for each hospital in the database. Group-average cost-to-charge ratio and hospital charges are available in the NIS database. All costs were adjusted to their 2010 dollar value using the national consumer price index.

### Exposure variables

The association of the outcome with the pertinent exposure variables was examined in a multivariate analysis. Age was a continuous variable. Gender, race (African American, Hispanic, Asian, or other, with Caucasian being the reference value), insurance (private insurance, self pay, Medicaid, with Medicare being the reference value), and income (defined as the median income based on zip code; income was divided into quartiles, with the lowest quartile being the reference value) were categorical variables.

The patient-level (Additional file 1: Table S1) comorbidities (categorical variables) were diabetes mellitus (DM), tobacco exposure, hypertension, hyperlipidemia, peripheral vascular disease (PVD), congestive heart failure (CHF), coronary artery disease (CAD), history of prior ischemic stroke, obesity, chronic renal failure (CRF), history of a TIA event, seizure disorder and coagulopathy. A categorical variable was used for the type of tumor resected (primary malignant tumor, metastasis, benign tumor, and other). The patient-level postoperative variables (categorical variables) were (Additional file 1: Table S1): treated hydrocephalus, hyponatremia, other neurologic complications, deep vein thrombosis (DVT), pulmonary embolism (PE), and acute renal failure (ARF). Lastly, hospitalization specific factors (continuous variables) were length-of-stay (LOS), number of procedures performed (NPx), and number of admission diagnoses (NDx).

The hospital characteristics used in the analysis as categorical variables included hospital region (West, South, Midwest, with Northeast being the reference value), hospital location (urban teaching, urban non-teaching, with rural being the reference value), and hospital bed size (medium, large, with small being the reference value). More information of the definitions of the various categories of hospital characteristics can be found at http://www.hcup-us.ahrq.gov/db/vars/nis_stratum/nisnote.jsp.

### Statistical analysis

Continuous variables were presented with the mean and standard deviation or median and interquartile range, whereas categorical values were presented as percentages. Continuous variables were compared using t-tests or Mann–Whitney test, and categorical variables were compared using Chi-square tests.

Initial analysis of cost data revealed significant positive skewness and kurtosis and linear regression analysis using cost resulted in a heteroskedastic variance of errors. In order to achieve normality the data were transformed using the natural logarithm (ln) transformation. Other transformations attempted included square root, cube root, and inverse transformation. These were not eventually used because the ln transformation provided the best fit for the data. The ln transformation significantly improved the skewness and kurtosis of the distributions (skewness = 0.12, kurtosis = −0.049). Normality was also assessed using histograms and Q-Q plots. The distributions of LOS, NDx, and NPx demonstrated significant positive skewness and kurtosis as well, and were also ln transformed before the analysis to achieve normality.

Our cohort was then randomized (1:1 randomization, in order to create two 50% sub-samples) to a derivation and a validation cohort. Subsequently, patients with missing values were removed from the cohort using listwise deletion. A parsimonious model was then developed in the derivation cohort by performing a stepwise linear regression including all the variables discussed previously. Dummy variables were created for non-binary categorical variables. The level of significance used for retention in the model was 0.05. No colinearity was observed by assessing tolerance and variance inflation factor (VIF). The regression diagnostics performed were the coefficient of determination (R^2^) and analysis of the residuals. Normality among the distribution of residuals was verified with histograms (Additional file 1: Figure S1 and S2), and P-P plots (Additional file 1: Figure S3 and S4). Further diagnostics included scatter plots of the standardized predicted values versus the standardized residuals, which revealed a random, symmetric distribution of values very close to zero (Additional file 1: Figure S5), therefore suggesting a linear fit of data.

The model created in the derivation cohort was applied on the validation cohort, the R^2^ was calculated and residual analysis was performed. The predicted values for the validation cohort were plotted against the observed values and goodness of fit was assessed. No heteroskedasticity was observed. For reporting purposes, we back transformed the data to demonstrate the percentage of the contribution of each variable to the cost value.

All probability values are the results of two-sided tests, and the level of significance was set at P < 0.05. Statistical analyses were performed using SPSS version 20 (IBM, Armonk, NY), XLSTAT version 2013.6.02 (Addinsoft, New York, NY).

## Results

### Patient characteristics

In the selected study period there were 36,433 patients (median age was 56.0 years, 53.3% females) undergoing CTR who were registered in NIS. Of these patients, 14,638 (40.2%) presented with primary malignant brain tumors, 9574 (26.3) with metastatic tumors, and 11414 (31.3%) with benign tumors (Table 1). Following 1:1 randomization and subsequent listwise deletion, derivation and validation cohorts were created. Randomization resulted in no significant differences in exposure factors between these two subgroups (Table 1).

**Table 1:** **Patient and hospital characteristics for patients undergoing craniotomy for tumor resection**

		**All patients**	**Derivation cohort**	**Validation cohort**	P-Value*
Sample size		36,433	18,218	18,215	
		**Median (IQR)**	**Median (IQR)**	**Median (IQR)**	
Age, median (IQR)		56 (23)	56 (23)	56 (22)	0.485
Length of Stay, median (IQR)		5 (6)	5 (6)	5 (6)	0.237
Number of Procedures, median (IQR)		2 (3)	2 (3)	2 (3)	0.060
Number of Diagnoses, median (IQR)		6 (5)	6 (5)	6 (5)	0.890
		**N (%)**	**N (%)**	**N (%)**	
Sex	F	19406 (53.26)	9660 (53.02)	9746 (53.51)	0.358
	M	17027 (46.74)	8558 (46.98)	8469 (46.49)	
Tumor type	Malignant primary	14638 (40.18)	7380 (40.51)	7258 (39.85)	0.197
	Benign	11414 (31.33)	5716 (31.38)	5698 (31.28)	0.847
	Metastatic	9574 (26.28)	4705 (25.83)	4869 (26.73)	0.051
	Other	807 (2.22)	417 (2.28)	390 (2.14)	0.338
Region	Northeast	7940 (21.79)	4017 (22.05)	3923 (21.54)	0.236
	Midwest	5299 (14.54)	2656 (14.58)	2643 (14.51)	0.852
	South	14259 (39.14)	7074 (38.83)	7185 (39.45)	0.229
	West	8935 (24.52)	4471 (24.54)	4464 (24.51)	0.939
Payer	Medicare	11274 (30.94)	5611 (30.80)	5663 (31.09)	0.549
	Medicaid	3951 (10.84)	2025 (11.12)	1926 (10.57)	0.096
	Private payer	18398 (50.50)	9241 (50.72)	9157 (50.27)	0.387
	Self-payer	1413 (3.88)	653 (3.58)	760 (4.17)	0.004
	Other	1397 (3.83)	688 (3.78)	709 (3.89)	0.565
		**All patients**	**Derivation cohort**	**Validation cohort**	P-Value*
		**N (%)**	**N (%)**	**N (%)**	
Race	Caucasian	28225 (77.47)	14148 (77.66)	14077 (77.28)	0.389
	African-American	2874 (7.89)	1382 (7.59)	1492 (8.19)	0.032
	Hispanic	3123 (8.57)	1576 (8.65)	1547 (8.49)	0.591
	Asian	978 (2.68)	505 (2.77)	473 (2.60)	0.301
	Other	1233 (3.38)	607 (3.33)	626 (3.44)	0.580
Location	Rural	875 (2.40)	433 (2.38)	442 (2.43)	0.756
	Urban, nonteaching	8028 (22.03)	3952 (21.69)	4076 (22.38)	0.115
	Urban, teaching	27530 (75.56)	13833 (75.93)	13697 (75.20)	0.103
Bedsize	Small	2130 (5.85)	1032 (5.66)	1098 (6.03)	0.139
	Medium	6062 (16.64)	3049 (16.74)	3013 (16.54)	0.617
	Large	28241 (77.51)	14137 (77.60)	14104 (77.43)	0.700
Quartiles of income	First quartile	7789 (21.38)	3916 (21.50)	3873 (21.26)	0.588
	Second quartile	8765 (24.06)	4407 (24.19)	4358 (23.93)	0.554
	Third quartile	9307 (25.55)	4654 (25.55)	4653 (25.54)	0.998
	Fourth quartile	10572 (29.02)	5241 (28.77)	5331 (29.27)	0.294
Postoperative complications		2932 (8.05)	1482 (8.13)	1450 (7.96)	0.541
		**All patients**	**Derivation cohort**	**Validation cohort**	P-Value*
		**N (%)**	**N (%)**	**N (%)**	
Comorbidities					
	Stroke	427 (1.17)	213 (1.17)	214 (1.17)	0.960
	TIA	20 (0.05)	13 (0.07)	7 (0.04)	0.180
	Diabetes	4725 (12.97)	2370 (13.01)	2355 (12.93)	0.820
	Obesity	1827 (5.01)	927 (5.09)	900 (4.94)	0.519
	Coagulopathy	971 (2.67)	490 (2.69)	481(2.64)	0.772
	Hyperlipidemia	7008 (19.24)	3453 (18.95)	3555 (19.52)	0.173
	Chronic Renal Disease	143 (0.39)	67 (0.37)	76 (0.42)	0.450
	Alcohol abuse	745 (2.04)	383 (2.10)	362 (1.99)	0.438
	CAD	1432 (3.93)	718 (3.94)	714 (3.92)	0.917
	Tobacco exposure	9312 (25.56)	4656 (25.56)	4656 (25.56)	0.993
	CHF	813 (2.23)	409 (2.25)	404 (2.22)	0.861
	Hypertension	14405 (39.54)	7137 (39.18)	7268 (39.90)	0.157
	Peripheral Vascular Disease	519 (1.42)	236 (1.30)	283 (1.55)	0.038
	Hydrocephalus	1955 (5.37)	990 (5.43)	965 (5.30)	0.564
	Hyponatremia	2309 (6.34)	1148 (6.30)	1161 (6.37)	0.777
	Seizures	2650 (7.27)	1338 (7.34)	1312 (7.20)	0.603
	Pulmonary embolism	1098 (3.01)	550 (3.02)	548 (3.01)	0.953
	DVT	225 (0.62)	120 (0.66)	105 (0.58)	0.316
	Acute Renal Failure	472 (1.30)	231 (1.27)	241 (1.32)	0.642

IQR: interquartile range; F: female; M: male; TIA: transient ischemic attack; CAD: coronary artery disease: COPD: chronic obstructive pulmonary disease; CHF: congestive heart failure; DVT: deep vein thrombosis.

Income quartiles were created with equal number of patients per quartile.

*Comparisons between groups were performed using the Mann–Whitney test and the Chi-square test as appropriate.

### Primary outcome

The mean and median hospitalization cost for patients undergoing CTR was $31,780 (95% CI, $31,518-$32,041) and $24,504 (Interquartile Range (IQR), $4,265-$44,743), respectively (Table 2a). Patients who were discharged home (Table 2b) had a lower hospitalization cost (median cost $21,735 (Interquartile Range (IQR), $15,514-$31,481)) in comparison to patients discharged to short-term care facilities (median cost $31,208 (Interquartile Range (IQR), $21,403-$47,662)).

**Table 2:** **Inflation-adjusted cost data**

**a.**											
**Total**				**Derivation cohort**				**Validation cohort**			
**Intracranial tumors**											
**Mean**	**95% CI**	**Median**	**IQR**	**Mean**	**95% C.I.**	**Median**	**IQR**	**Mean**	**95% C.I.**	**Median**	**IQR**
31,780	31,518-32,041	24,504	4,265-44,743	32,073	31,697-32,450	24,693	4,258-45,128	31,486	31,122-31,849	24,327	4,210-44,444
**b.**											
**Unfavorable discharge cohort**				**Favorable discharge cohort**				**P value**			
**Mean**	**95% C.I.**	**Median**	**IQR**	**Mean**	**95% C.I.**	**Median**	**IQR**				
41,446	40,898-41,993	31,208	21,403-47,662	26,504	26,292-26,716	21,735	15,514-31,481	<0.0001			

95% CI: 95% confidence intervals; IQR: interquartile range.

All cost values are inflation adjusted and have been converted to 2010 price values based on the consumer index.

### Model derivation

Several factors were included in our parsimonious model after stepwise linear regression (Table 3). Hospitals in the West and Midwest (45.5% and 16.1% more respectively, in comparison to the Northeast), African-Americans (3.9% more, in comparison to Caucasians), hydrocephalus (9.3% more), coagulopathy (8.4% more), post-operative neurologic complications (10.3% more), and higher income (6.2% more for the highest income quartile, in comparison to the lowest quartile) were associated with increased hospitalization cost. A 1% increase in LOS, and number of procedures was associated with a 0.5%, and 0.2% increase in cost, respectively. On the contrary, hospitals in the South (5.7% less, in comparison to hospitals in the Northeast), private insurance coverage (4.0% less, in comparison to coverage by Medicare), urban non-teaching hospitals (5.7% less, in comparison to rural hospitals), and medium bed size (8.8% less, in comparison to small hospitals) were associated with decreased cost. Our model could explain a significant portion of the variance in cost with an R^2^ of 0.62.

**Table 3:** **Percent change in hospitalization cost after craniotomy for tumor resection for every variable included in the final predictive model**

**Intracranial tumors**	
**Variable**	**% Change in cost**
Length of stay*	0.47
Number of procedures*	0.18
Midwest Region^1^	16.07
South Region^1^	−5.73
West region^1^	45.50
Medium Bedsize^2^	−8.79
Urban Nonteaching hospital^3^	−5.73
Private insurance^4^	−4.97
African American^5^	3.87
4th income quartile^6^	6.18
Hydrocephalus	9.31
Neurologic complications	10.30
Coagulopathy	8.44

* = Numbers represent percent change in cost for 1% change in the exposure variable (length of stay, number of procedures).

^1^in comparison to Northeast; ^2^in comparison to small bedsize; ^3^in comparison to rural hospital; ^4^in comparison to Medicare; ^5^in comparison to Caucasian; ^6^in comparison to the 1st (lowest) income quartile.

### Model validation

**Figure 2:**
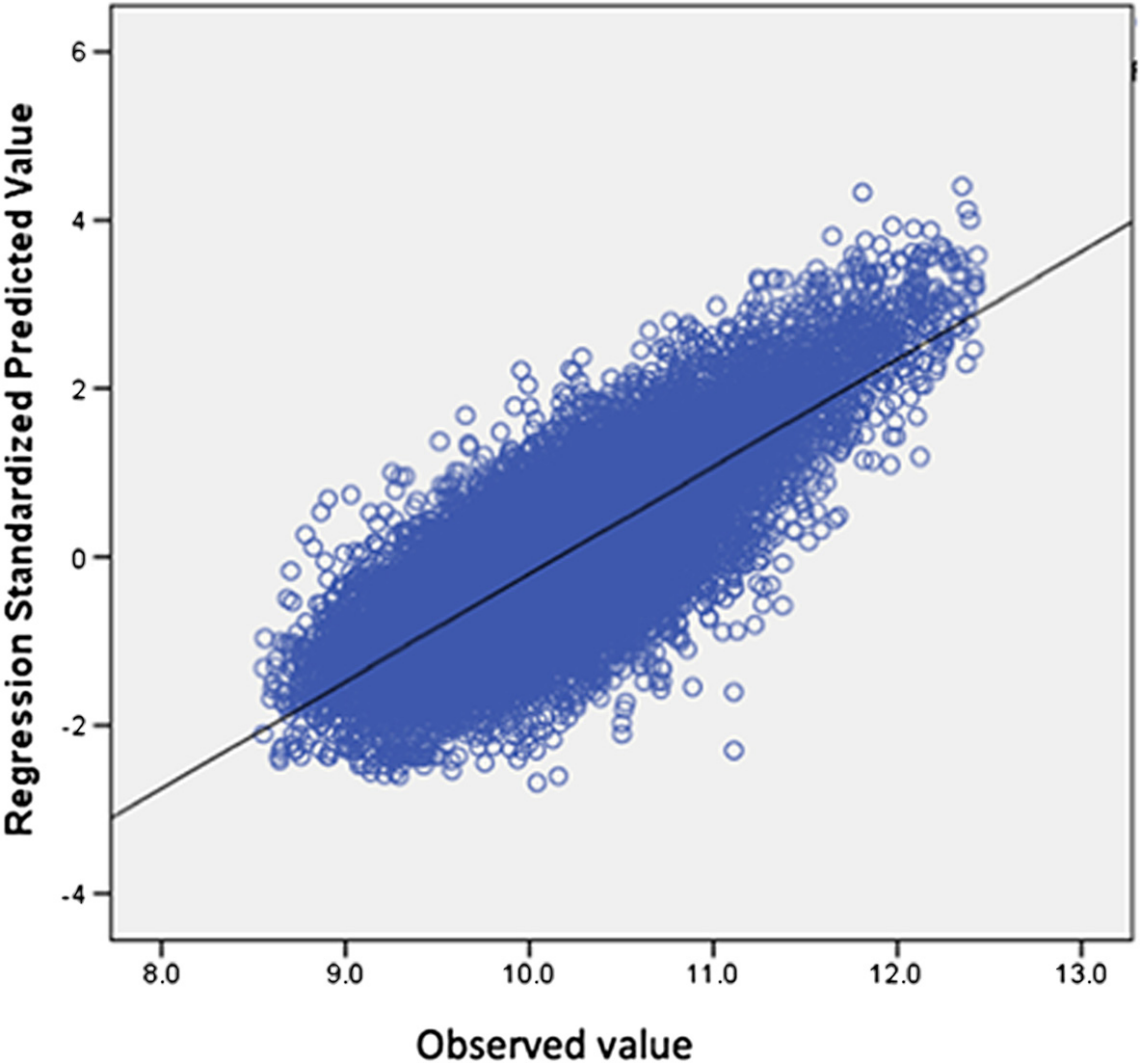
**Scatter plot demonstrating the association of the observed ln cost in the validation cohort and the predicted values of ln cost by the parsimonious model in patients undergoing craniotomy for tumor resection (Pearson’s rho = 0.77, P < 0.001).**

The model was validated in a random cohort of patients, and the final R^2^ did not differ more than 5% from the initial values (R^2^ = 0.60). There was very good association of the predicted values with the observed values in the validation cohort (Figure 2) (Pearson’s rho = 0.77, P < 0.001).

## Discussion

In this retrospective analysis of the NIS we developed a predictive model of hospitalization cost after CTR, and validated it in an independent cohort. The relative contribution of individual drivers of hospitalization cost after CTR have been identified. In a nation that spent $2.4 trillion on health care in 2008 alone, expenditures are under increasing scrutiny. A major component of the overall economic burden of healthcare is the initial hospitalization cost [14], especially in the setting of expensive, high-risk procedures, such as CTR. Although regulatory bodies have set general targets for cost containment [15], their applicability in specific procedures is still vague. This is particularly challenging, given the limited literature on factors associated with hospitalization cost variability. Although some studies have described the regional cost after CTR [11,12], there has been no particular focus on the identification of drivers of hospitalization cost, or the prediction of its magnitude.

To address this, we identified and quantified factors associated with cost variability after craniotomy for tumor resection. The major contributor to the observed changes in cost was length of stay, after controlling for patient and hospital characteristics. Although this finding is not surprising, since more cost is incurred with longer hospitalization, its relative contribution to the overall cost has not been studied before. Despite LOS being a major target for cost containment, the focus should be on excessively lengthy hospitalizations, not justified by patient comorbidities. The comorbidities contributing to increased LOS, in the setting of CTR, have been identified in prior studies [16], and should be taken into account to avoid penalizing the care of sicker patients.

Several other factors were identified. Most importantly, location of the hospital was crucial in determining the cost after CTR. The effect of regional variation on healthcare spending is widely recognized across medical specialties [17,18]. Geographic and racial disparities reflect the efficiency of local healthcare delivery systems, and the practices of individual physician groups. Cultural characteristics, litigation environment, and established local practices guide these trends. These result in differential resource utilization, which rarely translates in improved outcomes, whereas it is associated with higher cost. Minimizing regional disparities could contribute to reduced spending [17,18]. In regards to CTR, it appears that the West and Midwest were associated with significantly higher hospitalization cost in comparison to the Northeast, whereas the South was associated with lower cost. Additionally, we quantified the association of number of procedures with increased cost. Higher income was associated with higher cost, possibly secondary to the utilization of more expensive hospitals by this population. Lastly, hydrocephalus, other postoperative neurologic complications, and coagulopathy were identified as the most significant comorbidities contributing to higher cost. The magnitude of these associations was described.

The proposed predictive model for hospitalization cost after CTR was created and validated in a statistically rigorous way. Particular attention was given to normalizing the distribution of the primary outcome, and the continuous exposure variables in order to minimize errors in our regression analysis. In addition, residual analysis confirmed the linear fit of data. The diagnostics demonstrated that in both cohorts a significant portion of the cost variation could be explained by the variables included in our regression model. The model demonstrated good predictive ability in an independent validation cohort, with the predicted and observed values demonstrating good correlation.

Although our model cannot account for the full extent of cost variation, since it is limited by the data available through NIS, this is a first step in the direction of healthcare economics at the national level. It can be utilized as an adjunct in the cost containment debate, and the creation of data-driven policies. Our model can fuel further studies in the field and provide elements for the design of prospective investigations.

The present study has limitations common to administrative databases. First, indication bias and residual confounding could account for some of the observed associations. The 1:1 randomization of the cohort, and the validation of the model in an independent cohort aimed to minimize this bias. Second, several coding inaccuracies can affect our estimates, as in other studies involving the NIS. In addition, the number of admission diagnoses depends on the coding accuracy for each case and is therefore subject to the same limitations, which are inherent to administrative data. Third, the NIS during the years studied did not include hospitals from all states [13]. However, the creation of the 20% sample is done in such a way by HCUP that the hospitals included are still diverse with respect to size, region, and academic status. In addition, the structure of NIS, and the de-identification of the data do not allow patient follow up overtime in a longitudinal fashion, and therefore readmissions cannot be studied. Fourth, we are lacking the degree of neurologic impairment at presentation of the brain tumor patients. Fifth, some data categories were not available for all patients. To avoid the introduction of further bias we excluded those patients from any analysis. Sixth, we recognized postoperative neurologic complications based on one ICD-9 (997.00), which does not allow the identification of specific subcategories of complications. Seventh, causality is very hard to establish based on ecologic data. Our target was different though, and was focused on the identification of drivers of cost and the creation of a predictive model for it.

## Conclusions

The Nationwide Inpatient Sample (NIS) is a prospective all-payer, hospital discharge database that contains a representative sample of all inpatient admissions to nonfederal hospitals in the United States. By using this, several socioeconomic variables, as well as patient and hospital level factors associated with hospitalization cost variability after CTR were identified. Based on these data, a predictive model of cost after CTR was developed and validated in an independent cohort. Although the generalization of these predictions should be done with caution, the model can be utilized as an adjunct in the cost containment debate and the creation of data-driven policies. This can fuel further studies in the field and provide elements for the design of prospective investigations.

### Availability of supporting data

All supporting data are provided within this manuscript, tables, figures, and supplemental files

## Additional file

Additional file 1: Table S1. Coding definitions. **Table S2.** The 10 most common procedures performed during hospitalization for our cohort. **Figure S1.** Histogram of the distribution of standardized residuals in the derivation cohort. **Figure S2.** Histogram of the distribution of standardized residuals in the validation cohort. **Figure S3.** P-P plot demonstrating the association of predicted and observed residuals in the derivation cohort. **Figure S4.** P-P plot demonstrating the association of predicted and observed residuals in the validation cohort. **Figure S5.** Scatter plot of the standardized regression residuals versus the standardized predicted values for the derivation cohort.
