# Supplementary material for: Drivers of hospitalization cost after craniotomy for tumor resection: creation and validation of a predictive model
Source: BMC Health Serv Res. 2015 Mar 4;15:85. doi: 10.1186/s12913-015-0742-2 (PMC4351828; doi:10.1186/s12913-015-0742-2)

**Supplementary Material**

**Drivers of hospitalization cost after craniotomies for tumor resection: creation and validation of a predictive model**

Symeon Missios, M.D. ^1,*^, Kimon Bekelis, M.D. ^2,*^

^1^Department of Neurosurgery, Cleveland Clinic, Cleveland, OH

^2^Section of Neurosurgery, Dartmouth-Hitchcock Medical Center, Lebanon, NH

*These authors contributed equally to this work and are co-primary authors

**Table of Contents**

SUPPLEMENTARY TABLES…………………………………………………………………………………...3

Table S1. Coding definitions………………..…………...………………………………………………..………3

Table S2. The 10 most common procedures performed during hospitalization for our cohort…………………...4

SUPPLEMENTARY FIGURES………...………………………………………………………………………...5

Figure S1. Histogram of the distribution of standardized residuals in the derivation cohort……………..…...….5

Figure S2. Histogram of the distribution of standardized residuals in the validation cohort.……………….……6

Figure S3. P-P plot demonstrating the association of predicted and observed residuals in the derivation cohort..7

Figure S4. P-P plot demonstrating the association of predicted and observed residuals in the validation cohort..8

Figure S5. Scatter plot of the standardized regression residuals versus the standardized predicted values for the derivation cohort……………………………..…………………………………………..………….…………….9

| **Table S1. Coding definitions** | | |
| --- | --- | --- |
| **GROUP** | **CATEGORY** | **CODES** |
| **Diagnoses** | Brain tumors | **191.0, 191.1, 191.2, 191.3, 191.4, 191.5, 191.6, 191.7, 191.8, 191.9, 225.0, 225.1, 225.2, 237.5, 237.6, 237.6, 192.0, 192.1, 198.3, 200.5** |
| **Procedures** | Craniotomy | **01.51, 01.53, 0.59** |
| **Comorbidities** |  | |
|  | Transient Ischemic Attack | **435, 435.8, 435.9** |
|  | Stroke | **433.x1, 434.x1** |
|  | Seizures | **345.xx** |
|  | Myocardial Infarction | **410, 410.0-410.9, 411, 411.1, 411.89, 412, 413, 413.1, 413.9, 414, 414.2, 414.3, 414.4, 414.8, 414.9** |
|  | Tobacco exposure | **415.0, 416.8, 416.9, 491.0, 492.0, 494.0, 496.0**  **AND**  **305.1, V15.82, 989.84** |
|  | Congestive heart failure | **398.91, 402.11, 402.91, 404.11, 404.13, 404.91, 404.93, 428.0-428.9** |
|  | Diabetes Mellitus | **250.00-250.33, 250.40-250.73, 250.90-250.93** |
|  | Coagulopathy | **286.0-286.9, 287.1, 287.3-287.5** |
|  | Chronic Renal Failure | **403.11, 403.91, 404.12, 404.92, 585, 586, V42.0, V45.1, V56.0, V56.8** |
|  | Hypertension | **401.0-405.99** |
|  | Hyperlipidemia | **272, 272.0, 272.1, 272.2, 272.3, 272.4** |
|  | Obesity | **278.00, 278.01** |
|  | Alcohol abuse | **291.1, 291.2, 291.5, 291.8, 291.9, 303.90-303.93, 305.00-305.03, V113** |
|  | Peripheral vascular disease | **440.00-440.9, 441.2, 441.4, 441.7, 441.9, 443.1-443.9, 447.1, 557.1, 557.9, V43.4** |
| **Postoperative variables** |  | |
|  | Neurologic complications | **997.0x, 998.1x, 998.3x** |
|  | Treated hydrocephalus | **02.2, 02.32, 02.33, 02.34, 02.35** |
|  | Hyponatremia | **276.1** |
|  | Deep Vein Thrombosis | **451.1, 451.11, 451.19, 451.2, 451.81, 451.9, 453.2, 453.8, 453.9** |
|  | Pulmonary Embolism | **415.0, 415.1, 415.11, 415.13, 415.19, V12.51** |
|  | Acute Renal Failure | **584, 584.5, 584.6, 584.7, 584.8, 584.9, 583** |

| **Table S2. The 10 most common procedures performed during hospitalization for our cohort** | |
| --- | --- |
| Percentage | Procedure |
| 68.7 | **Craniotomy for excision of malignant tumor** |
| 31.3 | **Craniotomy for excision of benign tumor** |
| 8.14 | **CSF leak repair** |
| 4.85 | **Ventriculostomy** |
| 4.36 | **Transfusion of packed red blood cells** |
| 6.6 | **Cranioplasty** |
| 3.41 | **Cerebral angiogram** |
| 2.50 | **Postoperative intubation** |
| 1.88 | **Arterial catheterization** |
| 0.56 | **Central line placement** |

Figure S1. Histogram of the distribution of standardized residuals in the derivation cohort


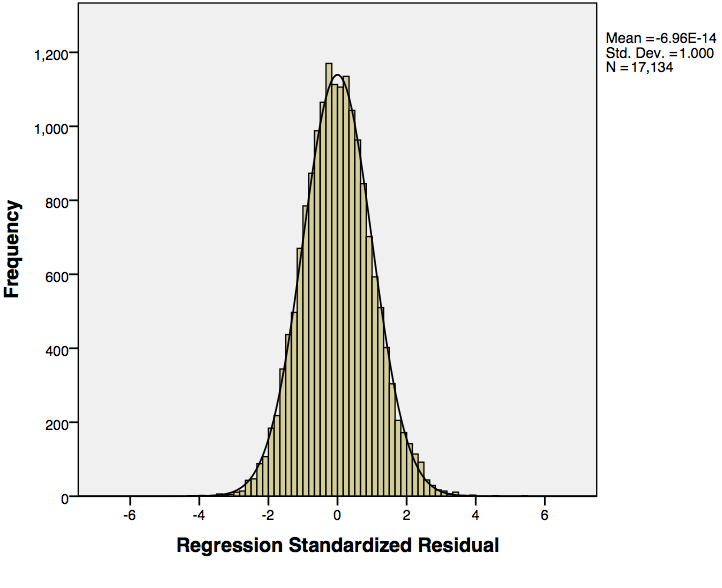


Figure S2. Histogram of the distribution of standardized residuals in the validation cohort


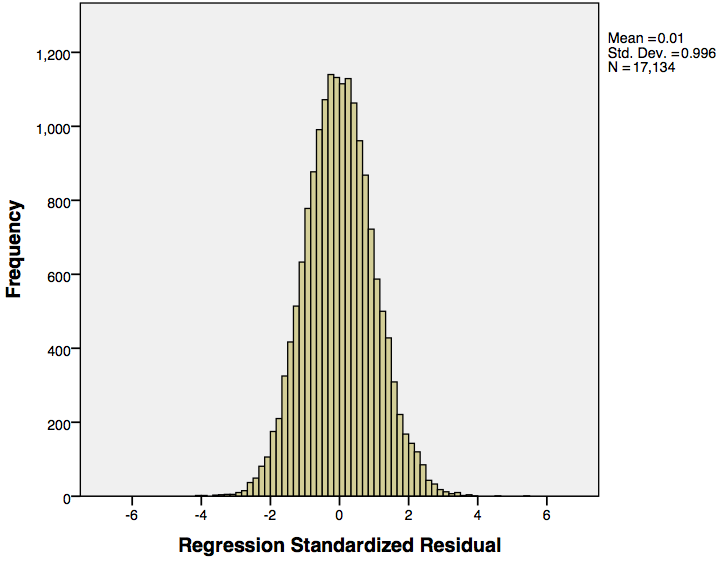


Figure S3. P-P plot demonstrating the association of predicted and observed residuals in the derivation cohort


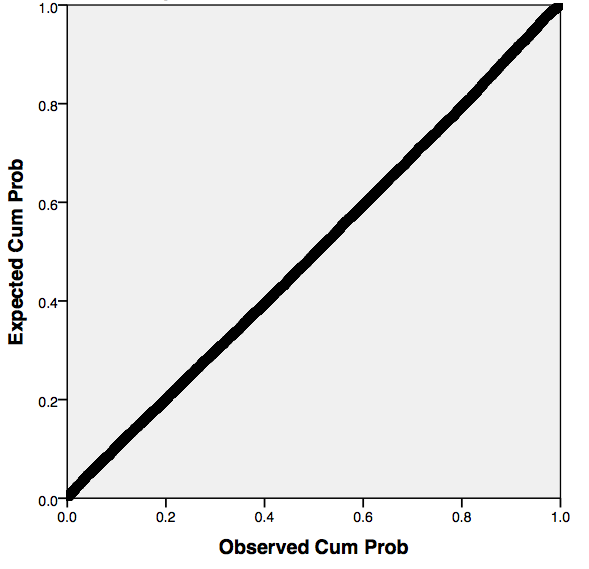


Figure S4. P-P plot demonstrating the association of predicted and observed residuals in the validation cohort


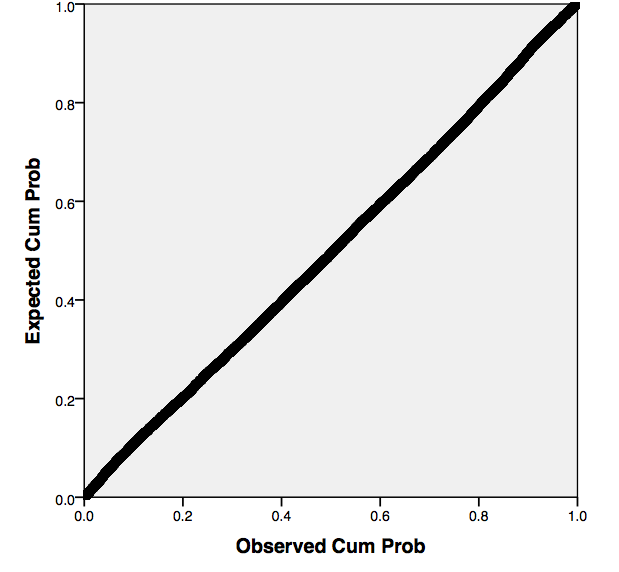


Figure S5. Scatter plot of the standardized regression residuals versus the standardized predicted values for the derivation cohort


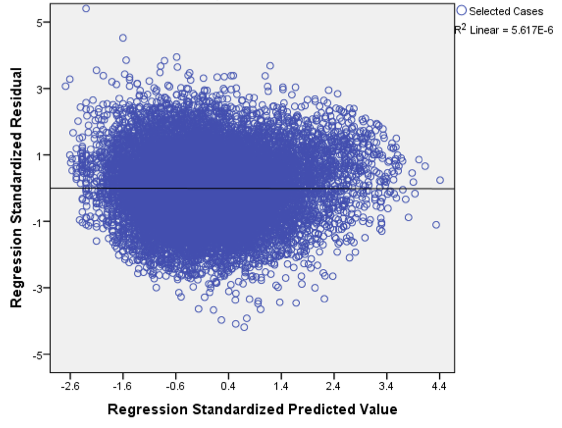

Supplement: Additional file 1: Table S1. — Coding definitions. Table S2. The 10 most common procedures performed during hospitalization for our cohort. Figure S1. Histogram of the distribution of standardized residuals in the derivation cohort. Figure S2. Histogram of the distribution of standardized residuals in the validation cohort. Figure S3. P-P plot demonstrating the association of predicted and observed residuals in the derivation cohort. Figure S4. P-P plot demonstrating the association of predicted and observed residuals in the validation cohort. Figure S5. Scatter plot of the standardized regression residuals versus the standardized predicted values for the derivation cohort. [file 12913_2015_742_MOESM1_ESM.doc]
